# O7.1. MIDBRAIN DOPAMINE NEURON ACTIVITY CONTROLS THE EFFECTS OF REPEATED KETAMINE ON STRIATAL DOPAMINERGIC FUNCTION

**DOI:** 10.1093/schbul/sby015.230

**Published:** 2018-04-01

**Authors:** Michelle Kokkinou, Oliver Howes

**Affiliations:** 1 Medical Research Council, London Institute of Medical Sciences; 2 Medical Research Council, London Institute of Medical Sciences, King’s College London

## Abstract

**Background:**

Schizophrenia is a chronic debilitating disorder which affects about 21 million people worldwide (WHO 2017). Elevated pre-synaptic striatal dopamine synthesis capacity is a robust neurochemical alteration seen in patients with schizophrenia compared to controls, with a large effect size Cohen’s d=0.79 (Howes et al., 2012). Ketamine, a non-competitive N-methyl-D-aspartate receptor (NMDAR) antagonist induces psychotomimetic effects in healthy human (Krystal et al., 1994, Stone et al., 2007) and exacerbates psychotic symptoms in patients with schizophrenia (Lahti et al., 1991). For these reasons, it has been used to model the neurochemical alterations seen in schizophrenia such as dopaminergic overactivity (Usun et al., 2013, Kokkinou et al., 2017). However, the effect of sub-chronic ketamine on dopamine synthesis capacity in vivo is not known. Here we investigated the effect of sub-chronic ketamine on striatal dopamine synthesis capacity in vivo using Positron Emission Tomography (PET) imaging and on locomotor activity in the mouse. Moreover, via a chemogenetics approach (Roth 2016) we explored the role of midbrain dopamine neuron activity in mediating ketamine-induced effects.

**Methods:**

All procedures were conducted under licence in accordance with the UK Animals (Scientific Procedures) Act of 1986. Mice received a sub-anaesthetic dose of ketamine or an equivalent volume of saline for five consecutive days. Locomotor activity was assessed in the open field test. Moreover, mice received a dynamic 3,4-dihydroxy-6-[(18)F]-fluoro-L-phenylalanine Positron Emission Tomography (PET) scan to assess striatal dopamine synthesis capacity in vivo. Data were analysed using an extended Patlak graphical analysis approach (Walker et al., 2013). Further midbrain dopamine neurons were transduced with an adeno-associated virus vector expressing Gi-coupled (hM4Di) inhibitory receptors under the control of the dopamine transporter (DAT) promoter in DATCre positive mice. Standard immunohistochemistry was used to label dopamine neurons and mCherry expression in dopamine neurons was confirmed using confocal microscopy. Two weeks following the stereotaxic injection of the viral construct, mice received clozapine N-oxide (CNO) to study the effects of inhibiting dopamine neuron firing on locomotor activity and striatal dopamine synthesis capacity in the sub-chronic ketamine model. Data were analysed by two-tailed independent samples t-tests, one-way ANOVA and repeated measures two-way ANOVA followed by Bonferroni post hoc tests where appropriate. p<0.05 was considered statistically significant.

**Results:**

Sub-chronic ketamine treatment significantly increased striatal dopamine synthesis capacity (p<0.05, effect size=1.2) and induced locomotor sensitization (p<0.01). hM4Di-mCherry viral construct was successfully transduced in midbrain dopamine neurons with over 98% specificity. Chemogenetic inhibition of midbrain dopamine neurons prevented the ketamine-induced elevation in striatal dopamine synthesis capacity (p<0.05, effect size= 0.64) and locomotor sensitization (p<0.05).

**Discussion:**

Our data show that sub-chronic ketamine results in the elevation in striatal dopamine synthesis capacity and locomotor sensitization and that these effects require midbrain dopamine neuron activation. Furthermore, our data are in support of the hypothesis that NMDA receptor hypofunction on GABAergic interneurons leads to disinhibition of glutamatergic projections and subsequently increase in dopamine neuron activity and dopamine synthesis capacity in projection targets such as the striatum.

